# Maturation of GABAergic Transmission in Cerebellar Purkinje Cells Is Sex Dependent and Altered in the Valproate Model of Autism

**DOI:** 10.3389/fncel.2018.00232

**Published:** 2018-07-30

**Authors:** Sébastien Roux, Ann Lohof, Yehezkel Ben-Ari, Bernard Poulain, Jean-Louis Bossu

**Affiliations:** ^1^Institut des Neurosciences Cellulaires et Intégratives (INCI)—CNRS, UPR 3212, Strasbourg, France; ^2^Sorbonne Université, CNRS UMR 8256, Biological Adaptation and Ageing, Paris, France; ^3^Neurochlore, Ben-Ari Institute of Neuroarcheology, Campus Scientifique de Luminy, Aix Marseille Université, Marseille, France

**Keywords:** cerebellum, Purkinje neuron, chloride gradient, autism, sexual dimorphism, GABA_A_ channels

## Abstract

Brain development is accompanied by a shift in gamma-aminobutyric acid (GABA) response from depolarizing-excitatory to hyperpolarizing-inhibitory, due to a reduction of intracellular chloride concentration. This sequence is delayed in Autism Spectrum Disorders (ASD). We now report a similar alteration of this shift in the cerebellum, a structure implicated in ASD. Using single GABA_A_ receptor channel recordings in cerebellar Purkinje cells (PCs), we found two conductance levels (18 and 10 pS), the former being dominant in newborns and the latter in young-adults. This conductance shift and the depolarizing/excitatory to hyperpolarizing/inhibitory GABA shift occurred 4 days later in females than males. Our data support a sex-dependent developmental shift of GABA conductance and chloride gradient, leading to different developmental timing in males and females. Because these developmental sequences are altered in ASD, this study further stresses the importance of developmental timing in pathological neurodevelopment.

## Introduction

Gamma-aminobutyric acid (GABA) is the main inhibitory neurotransmitter in the central nervous system. By opening GABA_A_ receptor channels and via chloride flow down its electrochemical gradient, GABA hyperpolarizes the membrane potential and thus reduces neuronal excitability in the mature nervous system. Intracellular chloride concentration (Cl^−^)_I_ is regulated by cation-chloride co-transporters that determine the strength of GABAergic inhibition (Rivera et al., 1999; Watanabe and Fukuda, 2015; Raimondo et al., 2017). If the intracellular chloride concentration is high enough that the equilibrium potential for chloride is positive compared to the resting membrane potential, GABA_A_ receptor activation can depolarize the cell above the action potential threshold, acting like an excitatory neurotransmitter (Ben-Ari et al., 1989; Ben-Ari, 2014). This regulation of (Cl^−^)_I_ has been reported in a wide range of animal species and brain structures; it is affected by many factors including sex steroids, BDNF and IGF-1 signaling (Galanopoulou, 2008; Tsutsui et al., 2011; Waddell and McCarthy, 2012; Ben-Ari, 2014; Watanabe and Fukuda, 2015). Immature neurons have lower (Cl^−^)_I_ than neurons of young adults, leading to depolarizing GABA actions which both activate voltage-gated calcium currents and allow NMDA receptor activation, underlying the well-known trophic actions of GABA in development (Ben-Ari, 2002, 2014; Witte et al., 2014; Raimondo et al., 2017). GABAergic signals also generate action potentials in many immature neurons, and the switch between excitatory and inhibitory GABA effects occurs at specific developmental time-points, depending on the neural structure and animal species. For example, the GABA “switch” occurs between E15.5 and E 17.5 in mouse spinal motoneurons (Allain et al., 2011), around P5 in the ventral horn of the rat spinal cord (Stein et al., 2004) and in ganglion and amacrine cells of the retina (Zhang et al., 2006), and from P8–P10 in mouse hippocampal pyramidal cells (Ben-Ari et al., 1989; Tyzio et al., 2007). Changes in the timing of the GABA switch, due to genetic mutations and/or environmental insults, are thought to contribute to neurodevelopmental disorders including autism (Ben-Ari et al., 1989; Ben-Ari, 2017).

GABA_A_ receptors are composed of 2 α subunits, 2 β subunits and 1ϒ subunit, which together form a central ion pore. Excluding splice variants and point mutations, 6 α isoforms, 3 β isoforms and 3 ϒ isoforms have been characterized, as well as some minor subunits (δ, ρ, ε, θ, π and ρ). In theory, therefore, a very large number of GABA_A_ receptor types may be found even in a single cell. The major adult isoform is generally accepted to be composed of α1, β2 and ϒ2 subunits. Whereas some subunits have a broad expression throughout the central nervous system, other subunits show a restricted cellular and subcellular localization (Sigel and Steinmann, 2012). The expression of GABA_A_ receptor subunits is developmentally regulated, notably with a developmental switch in α subunit expression associated with a slow-to-fast shift in the kinetics of GABA-mediated inhibitory postsynaptic potentials (Laurie et al., 1992; Fritschy et al., 1994). Thus, in thalamic reticular neurons, a postnatal switch in GABA_A_ receptor subunits from α5 to α3 is believed to play a role in the early development of the circuit (Pangratz-Fuehrer et al., 2016). Experiments using subunit expression in HEK cells or mouse fibroblasts show that single GABA channels currents have distinct opening conductance levels depending upon subunit composition (Mortensen and Smart, 2006).

Studies on developmental GABA shifts during normal development and in relation to autism spectrum disorders (ASD) have been done primarily in cortical structures. Yet abnormalities of the cerebellum and of cerebellar Purkinje cells (PCs) are frequently described in post mortem studies of humans with autism (Fatemi et al., 2012) as well as in the rodent valproate model of autism (Ingram et al., 2000). In addition, PCs undergo considerable post-natal development, including regression of climbing fiber multi-innervation and dramatic dendritic arbor expansion (Dusart and Flamant, 2012), allowing investigation of these important developmental processes after birth.

We have now measured the changes in somatic GABA_A_ channel properties and chloride gradient in PCs, comparing these changes in male and female mice, since sex differences in the GABA shift have been reported (Galanopoulou, 2008). We report that in normal and valproate-model male and female mice, the dominant GABA_A_ receptor channel shifts from high-conductance in the newborn to low-conductance in juvenile and adult mice. This shift in conductance parallels the GABA switch from depolarizing to hyperpolarizing, as it is sex dependent, being delayed in naïve females compared to males. Furthermore, the GABA switch is delayed in mice of both sexes after prenatal exposure to valproate, supporting a role of cerebellar dysfunction in the pathology of autism.

## Materials and Methods

### Mice

Pregnant C57 mice were injected intraperitoneally with 600 mg valproate sodium salt (*n* = 18) or saline (*n* = 15) at embryonic day 12.5 (Roullet et al., 2013). Either control pups or pups from valproate-treated dams were used to prepare acute cerebellar slices for patch-clamp experiments at ages between postnatal days 5 (P5) and 45 (P45). For the study of climbing fiber synapse elimination, either C57 or Swiss pups (because of large number of pups per litter) were used; no differences were found in synapse elimination between the two strains of mice. All procedures followed guidelines established by le Comité National d’ethique pour les Sciences de la Vie et de la Santé (EU Council Directive 2010/63/EU) and were approved by Institutional Animal Care and Use Committees (CREMAS, Comité Régional d’Ethique en experimentation animale de Strasbourg).

### Slice Preparation

Standard procedures were used to prepare 250-μm or 300-μm parasagittal slices from control or valproate-treated mice at P5–P45 following a protocol approved by the European and French guidelines on animal experimentation established by le Comité National d’ethique pour les Sciences de la Vie et de la Santé (EU Council Directive 2010/63/EU) and were approved by Institutional Animal Care and Use Committees (CREMAS, Comité Régional d’Ethique en experimentation animale de Strasbourg). Briefly, mice were killed by decapitation under isoflurane anesthesia. Brains were dissected in ice-cold artificial cerebrospinal fluid (ACSF) and sliced with a vibratome (Leica VT1200S) at 4°C. Slices were maintained for 30 min at 32°C in an interface chamber containing ACSF equilibrated with 95% O_2_, 5% CO_2_ and containing (in mM): NaCl 124, KCl 2.7, CaCl_2_ 2, MgCl_2_ 1.3, NaHCO_3_ 26, NaH_2_PO_4_ 0.4, glucose 10, ascorbate 4, then for at least 1 h at room temperature before being transferred to a superfusing recording chamber.

### Electrophysiological Recordings

Slices were transferred to a recording chamber on an upright microscope. The recording chamber was continuously perfused at room temperature with bath solution containing: (mM) NaCl 124, KCl 2.7, CaCl_2_ 2, MgCl_2_ 1.3, NaHCO_3_ 26, NaH_2_PO_4_ 0.4, glucose 10, pH 7.4, equilibrated with 95% O_2_, 5% CO_2_. For cell-attached recordings (in order to stabilized the resting membrane potential) the bath solution contained tetrodotoxin (TTX) 10^−5^ M and NBQX 10^−5^ M. In some experiments isoguvacine or NBQX were applied to the bathing fluid at a concentration of 10^−5^ M. For experiments recording climbing fiber currents, the ACSF contained 10^−4^ M picrotoxin.

Most electrophysiological experiments were performed on visually identified PCs using the patch-clamp technique in the cell-attached configuration. Electrodes were filled for single channels recordings with the following solution (mM): KCl 110, NaCl 2, MgCl_2_ 2, CaCl_2_ 2, HEPES 10, tetra-ethyl-ammonium-chloride (TEA) 20, TTX 10^−3^, CsCl_2_, 4-aminopyridine (4AP) 1, BaCl_2_ 1, Isoguvacine or Muscimol 10^−5^, pH 7.4 ; and for spiking activity with the following solution (mM): NaCl 124, KCl 2.7, CaCl_2_ 2, MgCl_2_ 1.3, NaHCO_3_ 26, NaH_2_PO_4_ 0.4, glucose 10, equilibrated at pH 7.4 with 95%O_2_, 5% CO_2_.

For experiments recording climbing fiber currents, patch pipettes were filled with a solution containing (mM): Cs-D-gluconate 120, biocytin 13, 10 HEPES, BAPTA 10, TEACl 3, Na_2_ATP 2, MgATP 2, NaGTP 0.2, pH 7.3, 290–300 mOsm. Climbing fiber currents were elicited by stimulation in the internal granular layer with a saline-filled glass pipette.

Signals were recorded and filtered at 5 kHz using an Axopatch 200A amplifier (Axon Instrument). Current and voltage signal were digitized at 50 kHz using a Digidata 1322A (Axon Instruments) prior to being recorded directly using Clampex (10.2) software. Analysis was performed off-line using Clampfit (10.2) software. Data were filtered before analysis with a cut off frequency of 1.5 or 1 kHz.

### Graphs, Fitting Procedures and Statistics

Sigma plot 12.5 software was used for graphic representations of the data, fitting procedures and statistical analysis. For channels conductance analysis the normality Shapiro-Wilk test, and equal variance test were used before running an un-paired Student’s *t*-test. Data were considered statistically significant when *P* < 0.05. * is used for *P* values between 0.05 and 0.01; ** for *P* values between *P* > 0.01 and 0.001. *** is used for *P* values < 0.001. The quality of the fit was determined using the prediction error.

## Results

### Two GABA_A_ Conductances in Neonatal PCs

**Figure 1:**
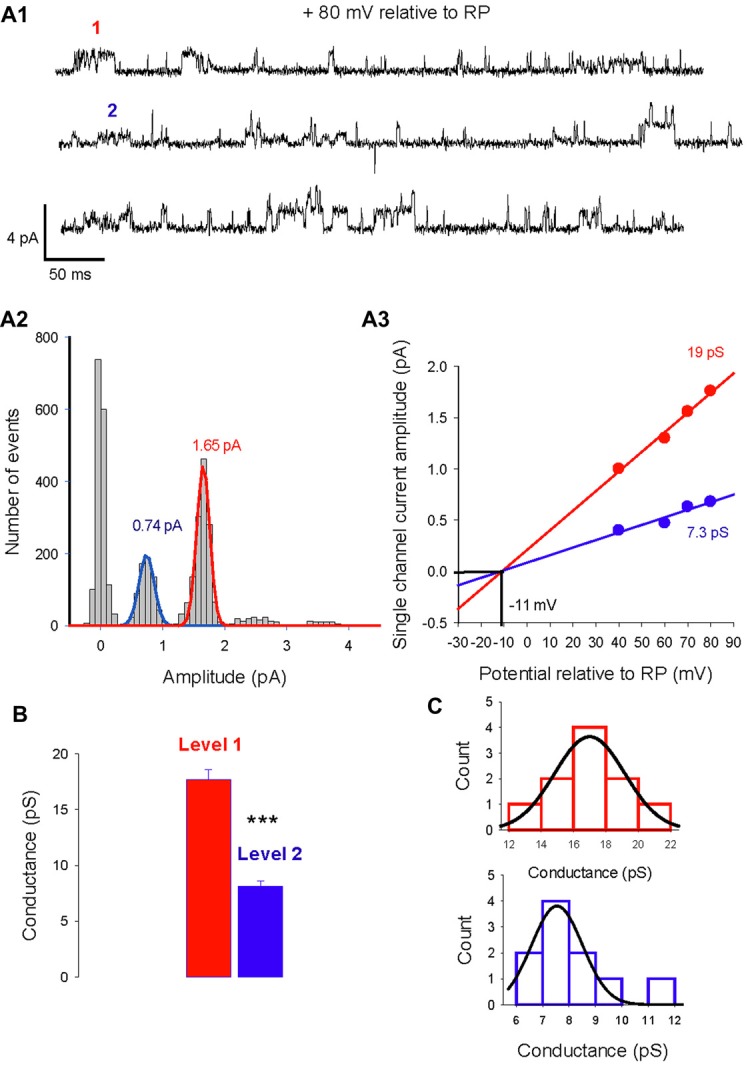
Two levels of type A gamma-aminobutyric acid (GABA_A_) receptor channel conductances can be recorded from the same Purkinje cell (PC) membrane patch. **(A1)** Current traces recorded in cell-attached conditions at a potential of 80 mV relative to resting potential (RP). Two amplitude levels of GABA_A_ receptor channels are seen (level 1 in red, level 2 in blue). **(A2)** Data from the same patch as in panel **(A1)** is illustrated in a current amplitude histogram. The distribution is fitted by two Gaussians, with peaks at 1.65 pA and 0.74 pA. **(A3)** The relation between single channel current amplitude and the potential relative to RP for the two conductance levels. Linear regressions give slope conductances of 19 pS (red) and 7.3 pS (blue). The reversal potential for the two conducting levels is −11 mV relative to RP. **(B)** Bar graphs of level 1 (red) and level 2 (blue) conductances recorded in 7 PC membrane patches. The mean value of level 1 is 17.7 ± 0.9 pA and differs significantly (***p < 0.001) from the mean value of level 2 (8.1 ± 0.5 pA). **(C)** Histogram distribution of the slope conductance of level 1 (red) and level 2 (blue). Each conductance distribution follows a normal distribution fitted by a Gaussian with a peak value of 17 pS for level 1 and 7.5 pS for level 2.

The single-channel properties of GABA_A_ receptors have been extensively studied in neuronal cultures and brain slices. In cerebellar granule cells, three main conductance (28, 17, 12 pS) have been characterized and are attributed to distinct GABA_A_ receptor subtypes (Brickley et al., 1999). We analyzed currents activated by isoguvacine or muscimol, two GABA_A_ receptor agonists, recorded from 108 cell-attached patches from PCs in acute cerebellar slices from male and female mice, between P5 and P45. In 10 patches, two conductance levels were recorded simultaneously (Figure 1). Figure 1A1 illustrates current traces showing two channels subtypes with a slope conductance of 19 pS and 7.3 pS, reversing at the same potential, indicating a similar ionic selectivity (Figure 1A3). The amplitude distribution in Figure 1A2 shows that the dominant channel conductance in this patch is level 1, and that it is not the result of simultaneous openings of two level 2 channels. The mean conductance slope value is 17.7 ± 0.9 pS for level 1 and 8.1 ± 0.5 pS for level 2 (Figure 1B, *n* = 10); these are significantly different (*P* < 0.001).

The histogram distribution of the slope conductance of level 1 (in red) and 2 (in blue) channels are illustrated in Figure 1C. For both channel types the amplitude distribution is normal and fitted with a Gaussian function but with distinct peak values, 17 pS for level 1 and 7.5 pS for level 2.

### GABA Conductance Switch During PC Development

**Figure 2:**
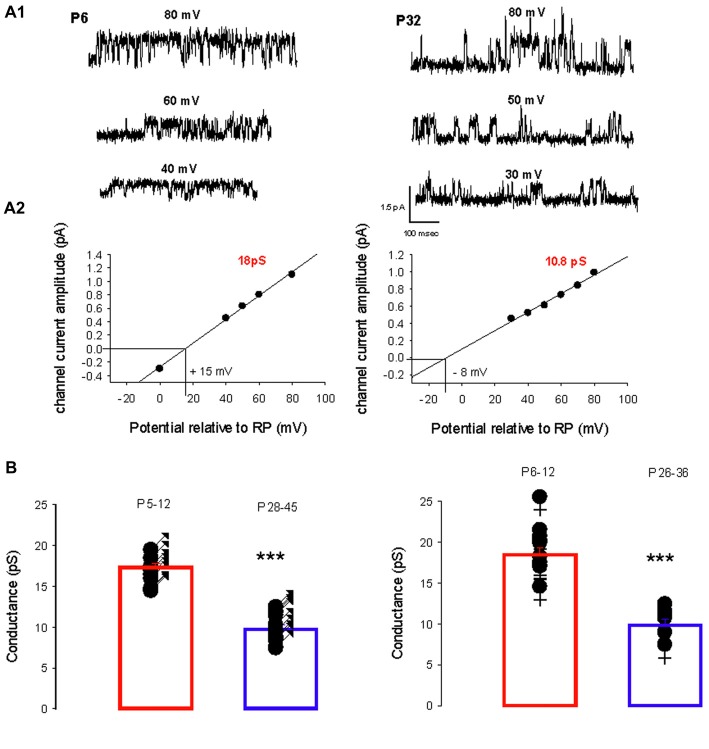
Developmental evolution of the main GABA_A_ receptor channel conductance. **(A1)** Current traces recorded in cell-attached conditions at different potentials relative to RP as indicated, from a female at P6 (left) and at P32 (right). **(A2)** Current-voltage relationship for the channel recorded at P6 (left) and at P32 (right). Linear regressions yield a conductance of 18 pS (level 1) and a reversal potential at +15 mV relative to RP for the channel recorded at P6; and a conductance of 10.8 pS (level 2) and reversal potential at −8 mV relative to RP for the channel recorded at P32. **(B)** The left panel shows the conductance of the dominant GABA_A_ receptor channel recorded from males between P5–P12 (*n* = 14) and P28–45 (*n* = 15). Each point represents an individual cell. The bar graphs show the mean values ± SD from P5–12 (red) and P28–45 (blue). These mean values (17.3 and 10.1 pS) are significantly different (***p < 0.001). The right panel illustrates the conductance of the dominant GABA_A_ receptor channel recorded from females between P6–P12 (*n* = 11) and P26–36 (*n* = 6). Each point represents an individual cell. The bar graphs show the mean values ± SD at P6–12 (red) and P26–36 (blue). These mean values (18.5 and 10.5 pS) are significantly different (***p < 0.001). The conductances at P5–12 in males and at P6–12 in females are not significantly different and neither is significantly different from the mean level 1 conductance. The conductances at P24–45 in males and at P26–36 in females are not significantly different and neither is significantly different from the mean level 2 conductance.

A switch in the dominant GABA_A_ receptor channel conductance occurs during development, from primarily level 1 in immature PCs (P5–12) to primarily level 2 in mature (P26–45) PCs in both sexes. Two recordings from PCs (female mice) illustrating this switch are shown in Figure 2A1. At P6, the slope conductance of the dominant GABA_A_ receptor channel is 18 pS, whereas at P24 it is 10.8 pS (Figure 2A2).

Figure 2B summarizes and compares the conductances of the dominant channels recorded in males (left panel) at P5–12 (newborn) and at P28–45 (young adult) and in females (right panel) at P6–12 and P26–36. In males the mean conductance of the dominant GABA_A_ channel switch from 17.3 ± 1.6 pS (*n* = 14) at P5–12 to 10.5 pS ± 1.7 pS (*n* = 15, *p* < 0.001) at P28–45; and in females from 18.5 ± 3.0 pS (*n* = 11) at P6–12 to 9.8 ± 8 pS (*n* = 6, *p* < 0.001) at P26–36. In newborn mice (both sexes), the mean conductance of the dominant GABA_A_ channel is not significantly different from the level 1 conductance illustrated in Figure 1; similarly, the mean conductance of the dominant GABA_A_ channel in young-adult mice (both sexes) is not significantly different from the level 2 conductance illustrated in Figure 1. Thus GABA_A_ channels in PCs switch from level 1 to level 2 during post-natal development.

### Sexual Dimorphism of the Chloride Gradient Shift

**Figure 3:**
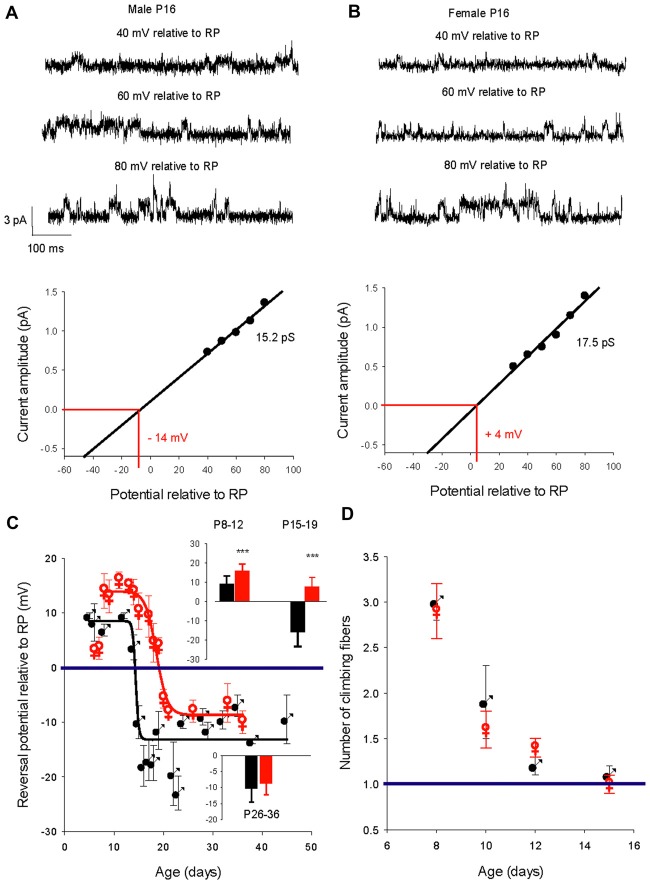
Developmental evolution of the GABA_A_ receptor channel reversal potential in males and females and climbing fiber synapse elimination. **(A)** Recordings from a P16 male. Current traces show the GABA_A_ receptor channel recorded from a PC membrane patch at three potentials as indicated on top of each trace. The I/V curve below, a linear regression of the data from this channel, gives a conductance of 15.2 pS and a reversal potential with an extrapolated value of −14 mV relative to RP. **(B)** Recordings from a P16 female. Current traces show the GABA_A_ receptor channel recorded from a PC membrane patch at three potentials as indicated on top of each trace. The I/V curve below, a linear regression of the data from this channel, gives a conductance of 17.5 pS and a reversal potential with an extrapolated value of + 4 mV relative to RP. Note that at the same post-natal age the GABA_A_ receptor channel reverses at a negative potential relative to RP in the male, but in the female reversal occurs at a positive potential relative to RP. **(C)** Left graph, evolution of the mean reversal potential of the dominant GABA_A_ receptor channel as a function of the age in males (black) and females (red). The data were best fit (R 0.98) by a sigmoidal hill function with four parameters (*f* = Y_0_ + a/(1 + exp(−(X − X_0_)/b)) between P5 and P48 in males (black line, a (max) = 22, b (slope) = −0.31, *X*_0_ = 14.4 and *Y*_0_ (min) = −13.5) and in between P8 and P36 in females (red line, *a* = 24.2, *b* = −1.25, *X*_0_ = 18.5, *Y*_0_ = −8.3). The horizontal line at 0 mV shows the depolarizing/hyperpolarizing switch. The bar graphs in inset compare the mean values of the reversal potential relative to RP in males (black) and in females (red) at P8–12, P15–19 and P26–36. A significant difference between males and females (***p < 0.001) was found at P8–12 and P15–19. **(D)** Right graph: regression of climbing fiber multi-innervation of Purkinje cells (PCs) is similar in males and females, indicating that this basic circuit maturation during this developmental period is not sex-dependent.

We then determined the reversal potential of the dominant GABA_A_ receptor channel relative to the resting membrane potential (RP) during PC development in males and females. Figure 3 illustrates current recordings obtained in PCs from a P16 male (Figure 3A) and a P16 female (Figure 3B). The reversal potential of the GABA_A_ receptor channel is determined by the linear regression used to fit the current/voltage (I/V) relationship of a single GABA_A_ receptor channel. At P16, the reversal potential is negative to RP (−14 mV) in males (Figure 3A, bottom panel), but positive to RP (+4 mV) in females (Figure 3B, bottom panel).

The changes in GABA reversal potential relative to RP in cerebellar PCs from males and females is illustrated in Figure 3C. The curve in males can be fitted with a sigmoidal function (four parameters, X_0_ 14.4 days). Between P5–P11 the reversal potential is stable, around +10 mV relative to RP; the reversal potential then rapidly reaches a negative value around P14 and stabilizes at P26 around −10 mV relative to RP (−10.3 ± 5.0 mV, *n* = 10 at *P* ≥ 26, see bottom graph in inset). Therefore, GABA exerts depolarizing effects in male PCs prior to P15 and hyperpolarizing effects thereafter.

With the exception of the earliest post-natal days, the developmental curve of the reversal potential in female PCs also follows a sigmoid pattern from P8 to P21 but with a systematically more depolarized value compared to males (Figure 3C, red symbols). Although the reversal potential is not significantly more negative between P5 and P7 in females than in males (+3.5 mV vs. +8.7 mV), it then becomes significantly (*p* < 0.001) more positive between P8 and P12 (+15.8 ± 3.6 mV, *n* = 11) than in males (+9.1 ± 4.1 mV, *n* = 8, see top graph in inset). From this higher plateau value, the reversal potential change over time is best fitted by a sigmoid curve (four parameters, X_0_ 18.7 days) and decreases to −8.5 ± 3.5 mV (*n* = 8), at P26 and older (see bottom graph in inset). These observations show that GABA exerts more depolarizing actions in female PCs than in male PCs during a period of nearly 2 weeks (P8 to P21). In addition, the GABA switch is delayed in females by 4 days (P18 vs. P14 in males), such that from P15–19 the reversal potential is negative relative to RP in males (−15.9 ± 7.4 mV, *n* = 17) but still positive in females (+7.6 ± 5 mV, *n* = 10; see top graph in inset).

An important step in cerebellar synaptic maturation during the early postnatal period is the refinement of climbing fiber synapses on PCs, from a multiple climbing fiber innervation to mono-innervation (Hashimoto and Kano, 2003). To determine possible links between GABA signaling and circuit refinement in terms of climbing fiber synapse elimination, we measured the number of climbing fiber synapses on each PC from males and females at different ages (Figure 3D). The progress of synapse elimination in both sexes was very similar, indicating that sex-dependent differences in GABAergic signaling does not disrupt this fundamental developmental process.

### The Developmental Switch of the Chloride Gradient Is Delayed in the Valproate Mouse Model of Autism

**Figure 4:**
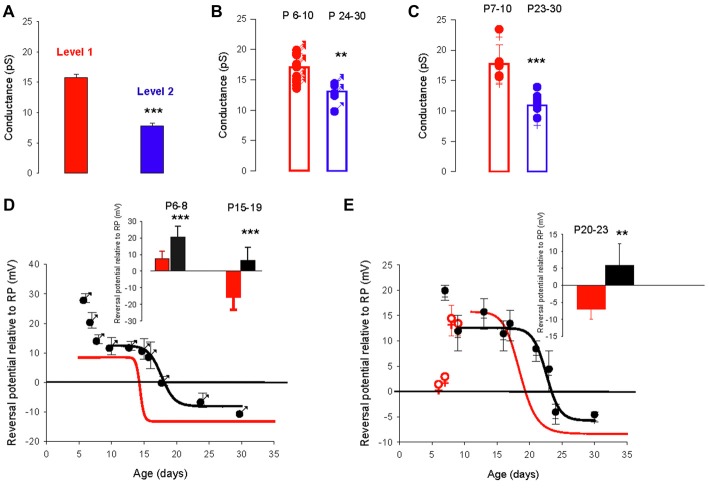
The valproate mouse model of autism alters developmental evolution of GABA_A_ channel reversal potential. **(A)** Mean slope conductance of the two conductance levels found simultaneously in five cell-attached recordings from PCs of male and female mice prenatally exposed to valproate. The level 1 (red) conductance (15.8 ± 0.4 pS) is significantly different from the level 2 (blue) conductance (7.8 ± 0.5 pS; ***p < 0.001). **(B)** Conductance of the dominant GABA_A_ receptor channel recorded in PCs from male valproate mice at P6–10 (*n* = 13) and at P24–30 (*n* = 5). Each symbol represents a single cell-attached recording. The bar graphs show the mean values ± SD between P6–10 (red) and between P24–30 (blue). These mean values (17.1 and 13.1 pS) are significantly different (**p = 0.002). **(C)** Conductance of the dominant GABA_A_ receptor channel recorded from female valproate mice at P7–P10 (*n* = 5) and at P23–30 (*n* = 7). Each symbol represents a single cell-attached recording. The bar graphs show the mean values ± SD at P7–10 (red) and at P23–30 (blue). These mean values (18.5 and 10.5 pS) are significantly different (***p < 0.001). The mean conductances at P6–10 in males and at P7–10 in females are not significantly different, and neither is significantly different from the level 1 channel conductance. The mean conductances at P24–30 in males and at P23–30 in females are not significantly different and neither is significantly different from the level 2 conductance. **(D,E)** Reversal potential of GABA_A_ receptor channels during development, in PCs recorded from male **(D)** and female **(E)** valproate mice. **(D)** In males, the depolarizing/hyperpolarizing switch is delayed in valproate animals (black symbols). Evolution of the GABA_A_ reversal potential with age (P5–P30). From P10 to P30 the data are best fit using a sigmoidal hill function with four parameters (black line, *a* = 21, *b* = −0.16, *X*_0_ = 17.7 and *Y*_0_ = −8). The curve in red represents the same curve but for control males. The inset bar graphs compare the mean values of the reversal potential relative to RP in control males (black) and valproate males (red) at P6–8 and P15–19. A significant difference between control males and valproate males (***p < 0.001) is detected. **(E)** In females, the depolarizing/hyperpolarizing switch is less altered in valproate animals (black symbols) Evolution of the GABA_A_ receptor channel reversal potential with age (P6–P30) in valproate females. Data are best fit by a sigmoidal function four parameters (black curve, *a* = 17, *b* = −0.28, *X*_0_ = 23 and *Y*_0_ = −5). The red curve is a sigmoidal fit of the evolution of the reversal potential in control females and the red open dots the mean value in control females not included in the fit by the red line. The bar graphs in inset compare the mean values of the reversal potential relative to RP in control females (black) and valproate females (red) at P20–23. A significant difference between control females and valproate females (**p = 0.003) is reported.

In the valproate rat model of autism, the developmental regulation of the chloride gradient, which determines the effects of GABA_A_ receptor activation on neuronal excitability, is disrupted in hippocampal neurons. As a result, the developmental excitatory-inhibitory switch of GABAergic effects is abolished in hippocampal neurons from valproate rats, with depolarizing actions from birth onwards (Tyzio et al., 2014). We used this same model in mice to analyze the developmental changes in GABA_A_ receptor channel conductance and the chloride gradient. We recorded 57 PC-attached recordings of GABA_A_ receptor channels from 35 males and 22 females from valproate-treated dams, at different postnatal ages. As in control mice, recordings from valproate mice revealed two conductance levels (level 1 mean value 15.8 ± 0.4 pS and level 2 mean value 7.8 ± 0.5 pS, *p* < 0.001) that could occasionally be recorded from the same patch (five patches, Figure 4A). Furthermore, as in control mice, PCs from valproate-treated male or female newborn mice show a dominant level 1 conductance channel, and PCs from juvenile and young adult PCs showed a dominant level 2 conductance channel. In males, the mean conductance switches from 17.1 ± 1.6 pS (*n* = 13) at P6–10, to 13.1 pS ± 1.8 pS (*n* = 5) at P24–30 (Figure 4B, *p* = 0.002). In females, the mean conductance switches from 17.7 ± 3.2 pS (*n* = 5) at P7–10, to 10.9 pS ± 1.6 pS (*n* = 7) at P23–30 (Figure 4C, *p* < 0.001). These conductance levels are not different between the sexes nor are they different from conductance levels 1 and 2 (Figures 1B, 4A) in control mice. Therefore, the conductance shift is not impacted in this ASD model.

However, the developmental change in the GABA_A_ receptor channel reversal potential was clearly more complicated in valproate-treated male mice (Figure 4D, black symbols) than in control male mice (red sigmoid, from Figure 3C). At P6–8 the reversal potential is significantly (*p* < 0.001, see graph in inset) more positive relative to RP (+20.6 ± 6.5 mV, *n* = 10) in valproate males compared to control males (+7.4 ± 4.6 mV, *n* = 9), decreases to a plateau value around +12 mV at P10, then reaches a negative value around P20 (sigmoid curve, *X*_0_ = P17.7 days). Thus, the switch from depolarizing to hyperpolarizing effect of GABA is delayed by 3 days in valproate male mice compared to control males. Therefore during a relatively long period, GABAergic currents are more depolarizing in valproate mice than in age matched control mice. Indeed, at P15–19 the mean reversal potential of the GABA_A_ channel is still positive in valproate mice (+6.6 ± 4.4 mV, *n* = 17 compared to −15.9 ± 7.4 mV, *n* = 11 in control male mice, *p* < 0.001, see graph in inset). These differences are less evident in females (Figure 4E). Yet at P7 in valproate female mice the reversal potential of GABA_A_ channels is around +20 mV relative to RP whereas it is about +5 mV relative to RP in control females. Furthermore, a comparison between the sigmoidal fit of the data obtained from valproate females (black curve, X_0_ 22.7 days) and the sigmoidal fit of the data obtained in control females (red curve, from Figure 3C) reveals a delay of the GABA switch of almost 4 days. As a consequence, at P20-P23 the mean reversal potential of the GABA_A_ channels is positive relative to RP (+5.8 ± 6.5 mV, *n* = 8) in valproate treated females but negative (−7.0 ± 3.0 mV, *n* = 8, *p* = 0.003) in control females (see inset Figure 4D).

### Sex Dependence of the GABA Conductance Shift: Parallel Development of GABA_A_ Channel Reversal Potential and Conductance

**Figure 5:**
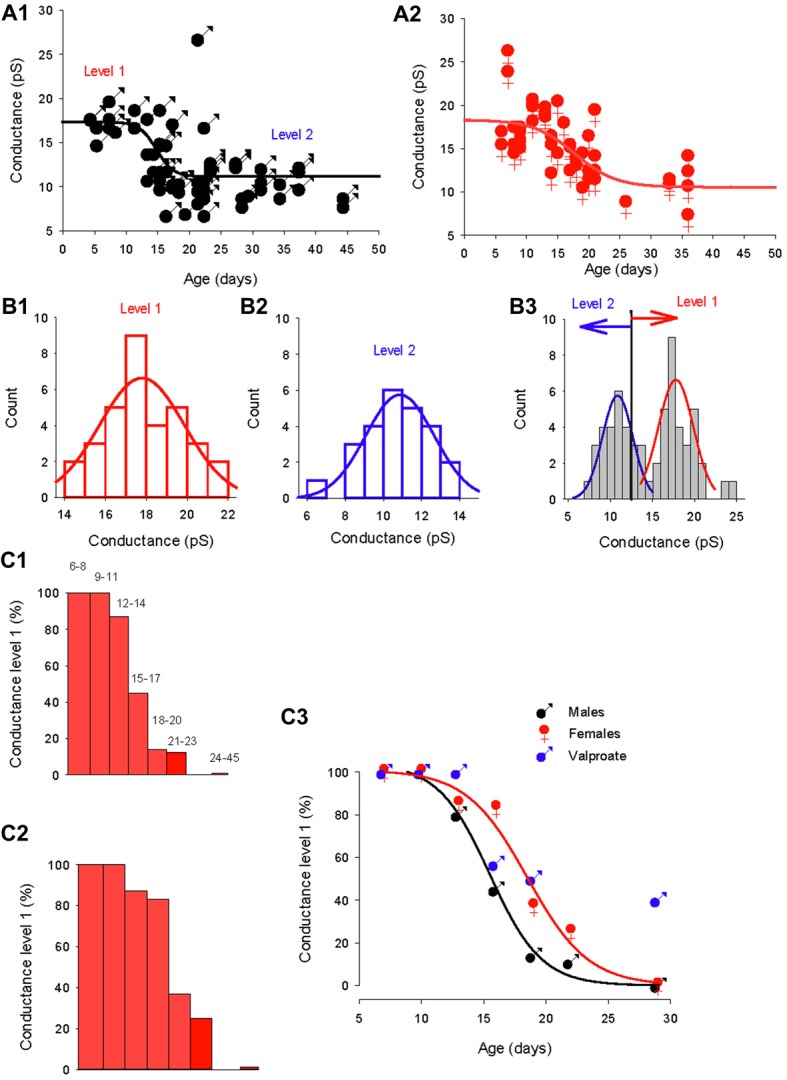
Distinct evolution of the GABA switch between conductance levels 1 and 2 in males, females and valproate-treated males. **(A1)** Dominant GABA_A_ channel conductance from level 1 to level 2 as a function of the post–natal age in males (each point represents a single cell). Data are best fit with a sigmoidal hill function with four parameters (black line, *a* = 6.2, *b* = −1.33, *X*_0_ = 14.7 and *Y*_0_ = 11.1). **(A2)** Dominant GABA_A_ channel conductance from level 1 to level 2 as a function of the post–natal age in females (each point represents a single cell). Data are best fit with a sigmoidal hill function with four parameters (red line, *a* = 7.8, *b* = −3.3, *X*_0_ = 17.7 and *Y*_0_ = 10.5). Note the presence of three channels with a conductance around 25 pS.** (B1)** Histogram distribution of the slope conductance of level 2 channels (pooled data from Figures 1B, 2B, 4A). The distribution is normal and fit by a Gaussian (blue line) with a peak value at 10.9 pA. **(B2)** Histogram distribution of the slope conductance of level 1 channels (pooled data from Figures 1B, 2B, 4A). The distribution is normal and fit by a Gaussian function (red line) with a peak value at 17.8 pA.** (B3)** Histogram distribution of the slope conductance of level 1 and level 2 channels (pooled data from **B1** and **B2**). To determine the limit of conductance between level 2 (blue line) and level 1 (red line) the Gaussian fits obtained in **(A1, A2)** were superimposed. The limit between levels 1 and 2 is 14 pS. Channels with conductances ≥14 pS are classified as level 1 channels and channels with conductances <14 pS are classified as level 2 channels. **(C1)** Data from male mice. The bar plots show the % of level 1 channels (conductance ≥14 pS) at increasing post-natal age ranges, as indicated on top of each bar.** (C2)** Data from female mice. The bar plots show the % of level 1 channels (conductance ≥14 pS) at increasing post- natal age ranges (the same range as in **C1**). **(C3)** Percentage of conductance level 1 as the function of the age in male mice (black symbols) female mice (red symbols) and valproate treated male mice (blue symbols). Data for control males (black curve) and females (red curve) are best fit using a sigmoidal function with the following parameters: *a* = 99, *b* = −1.9, *X*_0_ = 15.3 and *Y*_0_ = 3.2 for males; and *a* = 100, *b* = −2.42, *X*_0_ = 18.5, *Y*_0_ = 0.43 for females.

To determine whether the GABA_A_ channel conductance and the reversal potential switch occur in parallel, we compared the development of the GABA_A_ channels conductance in males and females (Figure 5). As a first approach, the slope conductance of the dominant GABA_A_ channel was plotted as a function of age in males (Figure 5A1) and females (Figure 5A2). The evolution of the conductance could be fitted in both sexes by a sigmoidal function (black and red lines) but with distinct parameters (slope = −1.33, *X*_0_ = 14.7 days for males and slope = −3.3, *X*_0_ = 17.7 days for females) indicating that the shift in conductance is slower and later in females compared to males. In both sexes, a single channel with larger slope conductance (25–27 pS, not included in the fit) was recorded in three patches and may indicate the presence of a rarely-occurring third conductance level of GABA_A_ receptor channel, as previously described for cerebellar granule cells (Brickley et al., 1999).

In order to quantitatively analyze the conductance switch, we determined the conductance ranges of the two levels. We first constructed separate histogram distributions of conductance levels 1 and 2 channels by pooling conductance values of GABA_A_ channels recorded in both sexes between P5–12 for conductance level 1 (Figure 5B1) and conductance values of GABA_A_ channels recorded at P28–45 in males and P26–36 in females for conductance level 2 (Figure 5B2). The distributions are fitted with distinct Gaussian functions, giving a peak of 17.8 pS for level 1 and 10.9 pS for level 2. Next, we combined the two distributions and associated fits (Figure 5B3), to show that the two fits intercept at a value of 13.9 pS. From this observation, we classified channels with a slope conductance of 14–22 pS as level 1, and channels with a slope conductance <14 pS as level 2.

Then, we determined the developmental alterations of level 1 channel percentage at different post-natal ages (Figures 5C1–C3). In both sexes the proportion of patches displaying dominant level 1 GABA_A_ receptor channel decreases with age; but the histograms in Figures 5C1,C2 show that this drop in level 1 conductance (defined as presence in less than 50% of patches) occurred earlier in males (P14; Figure 5C1) than in females (P17; Figure 5C2). Therefore, during a transitional period (around P15–20), PCs in females have a higher-conductance dominant channel than male PCs.

The evolution of the proportion of level 1 GABA_A_ channel as a function of age (Figure 5C3) is similar in both males and females to the evolution of the chloride reversal potential. Both curves are sigmoidal (four parameters) with similar X_0_ s (14.4 days for the level 1 plot and 15.3 days for the chloride reversal potential plot in males; and 18.5 days for both the level 1 plot and the chloride reversal potential in females). Interestingly the switch of GABA_A_ channel conductance is also altered in valproate male mice with 25% of level 1 channel dominant in young adult animals.

### Sexual Dimorphism of the GABA Excitatory/Inhibitory Shift

**Figure 6:**
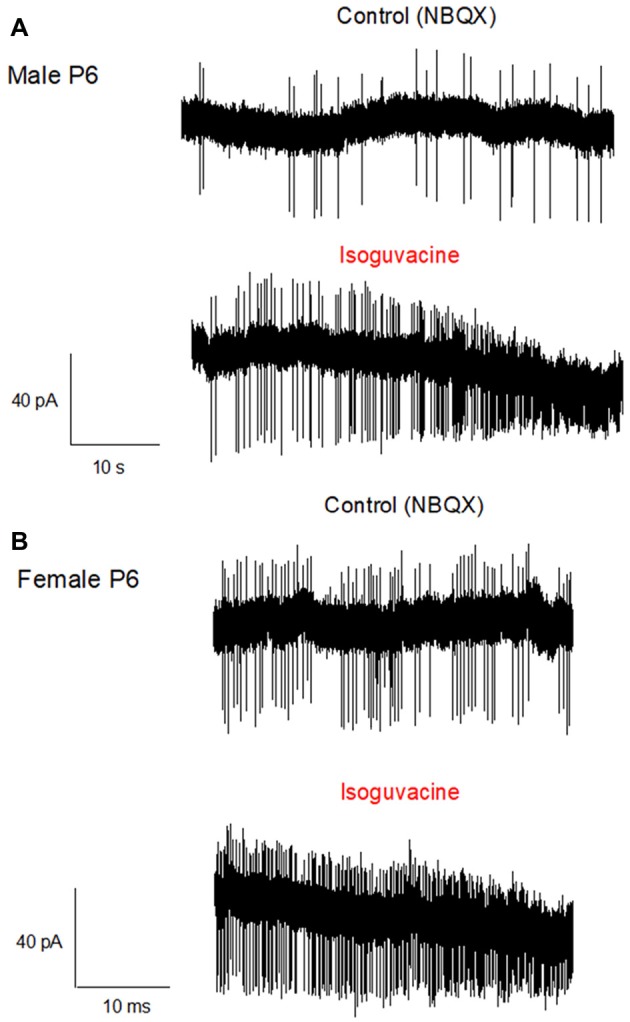
Activation of GABA_A_ receptors increases PC firing in P6 male and female mice. Cell-attached recordings (voltage clamp) of PC action potentials. **(A)** PC from a male at P6. The upper trace shows the control recording (in the presence of NBQX), and the lower trace shows the recording in presence of Isoguvacine (10^−5^ M). Isoguvacine application increases firing frequency and progressively reduced the spike amplitude. **(B)** PC from a female at P6. Upper trace, control conditions (in the presence of NBQX), lower trace in the presence of Isoguvacine. As in the male, isoguvacine increased firing frequency and progressively reduced spike amplitude.

**Figure 7:**
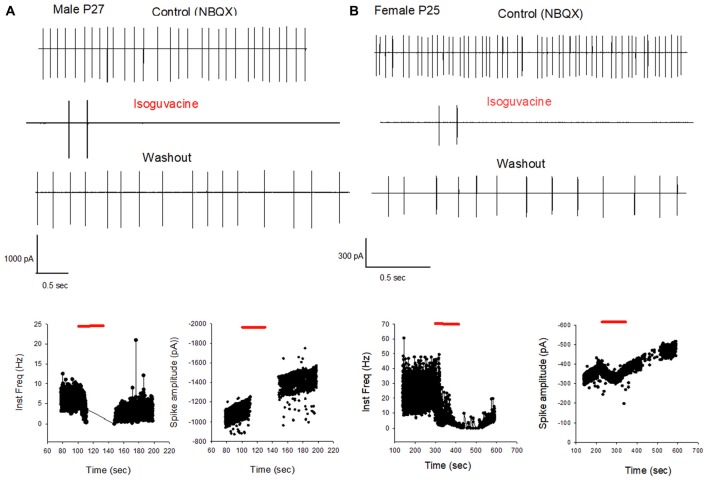
GABA_A_ receptor activation inhibits PC firing in young adult male and female mice. **(A)** Cell-attached recording (voltage clamp) of the spontaneous spiking activity of a PC from a male P27 mouse. Upper panel: current traces recorded in control conditions (in the presence of NBQX), in the presence of Isoguvacine, and after washout. Isoguvacine silences the cell, and activity recovers after washout. Lower panel: data from the same cell showing instantaneous action potential frequency (left) and action potential amplitude (right) over time. Isoguvacine application is indicated by the red line. The control spike frequency is about 7 Hz; isoguvacine abolishes the spiking activity of the cell; and this effect is reversible upon washout. Note that spike amplitude is not reduced before the silencing of the cell. **(B)** As in panel **(A)**, but from a female P25 mouse. Upper panel: current traces recorded in control conditions, in the presence of Isoguvacine, and following washout. Isoguvacine silences the cell, and activity recovers after washout. Lower panel: data from the same cell, showing instantaneous action potential frequency (left) and action potential amplitude (right) over time. Isoguvacine is applied as indicated by the red line. The control spike frequency is around 30 Hz. In presence of isoguvacine the spiking activity of the cell is almost abolished, but the spike amplitude is not altered.

Finally, we determined possible links between the chloride driving force and the excitatory or inhibitory actions of the GABA_A_ agonist isoguvacine on PC firing, with or without NBQX. Similar effects of isoguvacine were observed in the presence of NBQX suggesting that glutamatergic neurotransmission was not involved (not shown; *n* = 80). When the reversal potential of the GABA_A_ channel was positive to RP (at P6), isoguvacine increased the firing rate in males (Figure 6A, *n* = 3 out of 5) and in females (Figure 6B, *n* = 3 out of 6). This effect was accompanied by a progressive decrease in the size of the spikes due to the sodium channel inactivation produced by a strong depolarization. In the remaining recordings, PCs were silent and isoguvacine applications led to a depolarization. In older PCs, when the reversal potential of the GABA_A_ channels is negative to RP, isoguvacine decreased spontaneous activity of PCs, without altering the spike amplitude in both males (P27; Figure 7A) and females (P25; Figure 7B).

**Figure 8:**
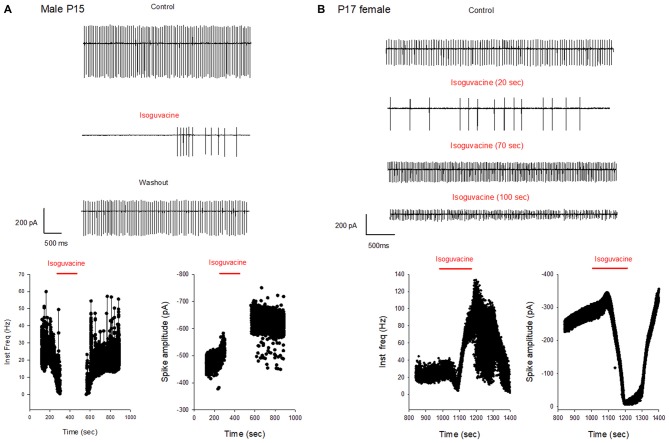
Different effects of GABA_A_ receptor activation in males and females at P15–P17. **(A)** Cell-attached recording (voltage-clamp) of spontaneous spiking activity of a PC from a P15 male mouse. Upper panel: current traces recorded in control conditions, with isoguvacine, and after washout. Regular action potential firing is reduced in the presence of isoguvacine and recovers after washout. Lower panel: data from the same cell showing instantaneous action potential frequency with time (left) and action potential amplitude with time (right). Isoguvacine application is indicated by the red line. Control firing frequency is about 30 Hz; isoguvacine abolishes firing, and this effect is reversible. Note that the abolition of the discharge is not linked to a decrease in spike amplitude. **(B)** As in panel **(A)**, but from a P17 female mouse. Upper part: current traces recorded in control conditions, with isoguvacine, and after washout. Regular action potential firing is reduced in the presence of isoguvacine and recovers after washout. Lower graphs, from the same cell, show instantaneous action potential frequency with time (left) and action potential amplitude with time (right) Isoguvacine application is indicated by the red line. Control firing frequency is about 30 Hz; with isoguvacine application, a brief inhibition of the firing frequency is followed by an increase, up to 100 Hz; this effect is reversible. Isoguvacine also reversibly decreased spike amplitude.

Additional experiments confirmed the sex and age difference in the actions of isoguvacine. In males at P15, after 2 min isoguvacine application, PC firing was blocked by 75 ± 35% in all cells tested (*n* = 4; example in Figure 8A), and completely blocked (100%) for PCs recorded from P16 to P29 (*n* = 14; example in Figure 7A). However in females at P17, 2 min of isoguvacine increased PC firing frequency by 220 ± 161%, accompanied by a reduction in spike amplitude in 5 out of 13 PCs (example in Figure 8B), sometimes preceded by a temporary reduction in the firing frequency (*n* = 2, including the example in Figure 8B). Isoguvacine had no effect on firing frequency in two PCs from female mice, and inhibited firing frequency in seven of these PCs. The excitatory effect of isoguvacine continued to be seen in female PCs (*n* = 2 out 5) until P19; then at P20–25 isoguvacine had only inhibitory effects (87% ± 8% inhibition, *n* = 6).

**Figure 9:**
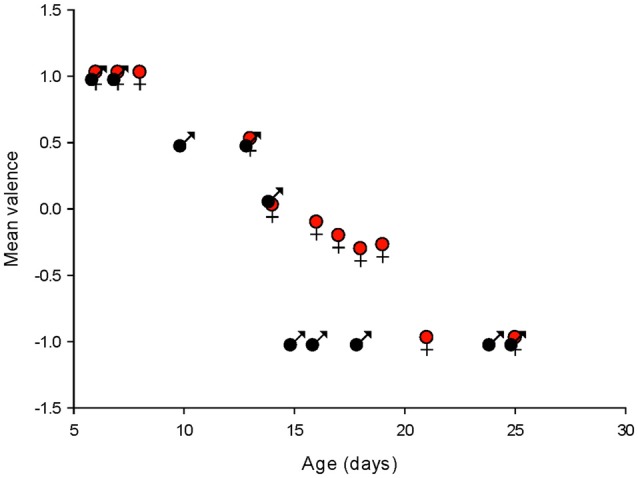
Summary of changes in spontaneous action potential frequency following GABA_A_ receptor activation by isoguvacine, in males and females. The mean valence of the isoguvacine effects is plotted as a function of the post-natal age in male and female mice.

To summarize the effects of isoguvacine on spontaneous PC, firing we assigned a value of +1 when isoguvacine produced an increased firing frequency, 0.5 when the increase was preceded by a short inhibition of firing, 0 when isoguvacine had no effect and −1 when isoguvacine inhibited firing. Figure 9 shows the mean valence as a function of age for PCs from male and female mice, and clearly illustrates the different time course of the shift to inhibitory isoguvacine effects between male and females.

## Discussion

### Development of GABA_A_ Receptor Channel Subunit Composition in Cerebellar PCs

Somatic GABA_A_ receptor channels in adult vertebrate neurons have complex gating and multiple conductance states ranging from 7 to 36 pS, with a single dominant conductance state in each cell type (Newland et al., 1991; Robello et al., 1998; Brickley et al., 1999; Mortensen and Smart, 2006). We found three main conductance levels in PC, with conductances of 7–12 pS, 15–18 pS, and occasionally 25–28 pS. Similar conductance values are observed in adult cerebellar granule cells, but with a shift of the dominant conductance from medium (15–18 pS) to small (7–12 pS) during maturation. This shift likely reflects a reorganization of GABA_A_ channel subunit composition (Moss et al., 1990; Verdoorn et al., 1990; Mortensen and Smart, 2006). Although it is difficult to equate GABA conductance levels with specific subunits combinations (Brickley et al., 1999), it is likely that in young-adult PCs, receptors lack the γ subunit (Verdoorn et al., 1990; Fisher and Macdonald, 1997; Amato et al., 1999; Hörtnagl et al., 2013). The dominant medium conductance level (18 pS) in newborn PCs suggests the presence of an α1β1 GABA_A_ receptor at this developmental stage (Moss et al., 1990). The very rare occurrence of the 25–28 pS conducting level is may be related to a low level of expression of the γ2 subunit (Mortensen and Smart, 2006). Our observations suggest a subunit reorganization during development (but see Nadler et al., 1994; Haghir et al., 2013) but further experiments are required to determine if this is associated with changes in IPSCs kinetics.

### Sexual Dimorphism of GABA Driving Force in Wild Type Mice and in the Valproate Model of Autism

In a wide range of brain structures, the polarity of GABA_A_ receptor effects is regulated by the expression and activity of two chloride membrane transporters: NKCC1 and KCC2. NKCC1 is expressed in immature neurons and actively transports chloride ions into the cell; KCC2 is expressed in mature neurons and actively transports chloride ions out of the cell. This changes in transporter expression leads to depolarizing effects of GABA in immature neurons and hyperpolarizing effects in mature neurons (Ben-Ari, 2014; Watanabe and Fukuda, 2015; Raimondo et al., 2017) with a developmental switch around birth (Tyzio et al., 2014; Watanabe and Fukuda, 2015). However, PCs do not express NKCC1 (Mikawa et al., 2002), suggesting that alternative mechanisms are required to regulate chloride homeostasis, including chloride channels (Zhang et al., 2015; Rahmati et al., 2016) and KCC2. The developmental GABA switch occurs in PCs at the end of the second post-natal week (see also Eilers et al., 2001), when profound morphological and functional alterations occur in PCs (Dusart and Flamant, 2012). The timing of the switch is strongly dependent on the specific cerebral structure; it is similar in the antero-ventral cochlear nucleus (Song et al., 2012) and substantia nigra pars reticulata (SNR; Kyrozis et al., 2006), and earlier in other brain structures (Stein et al., 2004; Tyzio et al., 2007; Allain et al., 2011; Witte et al., 2014). Our results also suggest that the regulation of chloride homeostasis is sex dependent, since it is delayed in female mice. A similar sex dependence of chloride homeostasis has been observed in the substantia nigra (Galanopoulou, 2008).

The mechanisms underlying these sex differences are poorly understood. The testosterone surge in males that occurs during late gestation and shortly after birth (Dean and McCarthy, 2008) has been suggested to promote the expression and the activity of NKCC1 and to depress the synthesis and activity of KCC2 (Waddell and McCarthy, 2012), and thus make the GABA switch occurs later in males than in females. This effect has been shown in hippocampal cultures (Nuñez and McCarthy, 2007), SNR neurons in acute slices (Kyrozis et al., 2006), and embryonic hypothalamic neurons in culture (Mir et al., 2017). However, the GABA switch in PCs is delayed in females, rather than in males, despite this testosterone effect on KCC2, suggesting that alternative mechanisms must be present. PCs synthesize estradiol from cholesterol in the neonatal period and the enzymes involved in this synthesis are developmentally regulated with a different profile in males and in females (Dean and McCarthy, 2008). In PCs, estradiol promotes BDNF-mediated dendritic growth, spinogenesis and synaptogenesis during neonatal life (Tsutsui et al., 2011), and BDNF inhibits KCC2 activity (Huang et al., 2013). Furthermore, the development of the cerebellar expression of insulin receptors (IR) and insulin-like growth factor-1 receptors (IGF-1R) differs in males and females. IR and IGF-1R expression increase between P0–P7 and is down regulated at P14 in males, whereas in females IR and IGF-1R are stable between P0–P7 and up-regulated at P14 (Haghir et al., 2013). IGF-1 accelerates the developmental switch between NKCC1 and KCC2 chloride transporters in the visual cortex (Baroncelli et al., 2017). Collectively, these observations converge to suggest multiple mechanisms to explain the differences between chloride homeostasis regulation in males and females.

Using the valproate mouse model of autism, we observed that the chloride reversal potential in P5–6 valproate males is more positive than in controls (by about 10–20 mV), and that the GABA switch is delayed by 3–4 days in both males and females, extending this experimental model to the cerebellum. Increased excitatory-inhibitory ratio is suggested to underlie the pathogenesis of autism and successful clinical trials have shown that the NKCC1 antagonist bumetanide, which restores low (Cl^−^)_I_ and GABAergic inhibition, also attenuates the severity of autism (Rubenstein and Merzenich, 2003; Lemonnier and Ben-Ari, 2010; Tyzio et al., 2014; Lee et al., 2016; Uzunova et al., 2016; Lemonnier et al., 2017). It is now accepted that the cerebellum is involved in higher order functions (Schmahmann and Sherman, 1998) including perceptual processes (Baumann et al., 2015), in addition to its roles in balance, posture, and motor control. The cerebellum has been implicated in many psychiatric disorders (Phillips et al., 2015) and cerebellar abnormality is associated with ASD (Tsai et al., 2012). Several reports in individuals with autism or animal models of autism describe alterations in PC density and properties (Skefos et al., 2014; Tsai, 2016). Thus, chloride gradient modifications and alterations in the GABA switch in cerebellar PCs that we show here may be particularly relevant for further studies of autism and the role of the cerebellum.

### Parallel Development of GABA_A_ Receptor Channel Subunit Composition and Chloride Gradient

Our data show that the kinetics of the shift of the dominant conductance levels during development, from medium (15–18 pS) to small conductance (7–12 pS), are similar to the kinetics of the development of the chloride gradient. Both phenomena followed a similar sigmoidal process, are delayed in females, and are altered in valproate mice, suggesting interactions between the subunit composition of GABA_A_ channels and intracellular chloride concentration (Succol et al., 2012).

Interestingly, in PCs, it has been suggested that an interaction between KCC2 and a specific subunit of the GABA_A_ receptor represents a fundamental mechanism of regulation of GABAergic synapses (Huang et al., 2013). Furthermore, membrane expression of the β3 GABA_A_ receptor subunit at different postnatal developmental stages is observed in rats exposed prenatally to valproic acid (Li et al., 2017). Finally, alterations in the efficacy of neuronal inhibition mediated by GABA_A_ receptors containing β3 subunits have been implicated in autism (Vien et al., 2015). Collectively, these observations raise the possibility of parallel convergent developmental alterations of GABAergic signals, linking chloride gradient and subunit composition.

## Conclusions and Perspectives

The conductance properties of GABA_A_ receptors as well as the chloride equilibrium potential and the polarity of GABA effects in PCs are developmentally regulated in parallel, occurring later than in other brain structures, which is coherent with the delayed development of cerebellar PCs to the postnatal period. The switch is also delayed by 4 days in females, indicating sexual dimorphism in keeping with recently reported sex differences in synaptic excitation, inhibition and intrinsic properties (Mercer et al., 2016). Finally, we show that the valproate model of autism produced a shift in the chloride gradient in developing PCs from males and females, providing important potential implications for the cerebellum in the pathology of autism.

## Author Contributions

SR, AL, YB-A, BP and J-LB wrote the manuscript. SR, AL and J-LB designed the research, performed the experiments and analyzed the data.

## Conflict of Interest Statement

YB-A is CEO of Neurochlore, a biotech company devoted to treat autism. The remaining authors declare that the research was conducted in the absence of any commercial or financial relationships that could be construed as a potential conflict of interest.
